# Supplementary material for: Timeless couples G‐quadruplex detection with processing by DDX11 helicase during DNA replication
Source: EMBO J. 2020 Jul 23;39(18):e104185. doi: 10.15252/embj.2019104185 (PMC7506991; doi:10.15252/embj.2019104185)
Supplement: Supplementary file 1 — Appendix [file EMBJ-39-e104185-s001.docx]

**Appendix for**

**Timeless couples G-quadruplex detection with processing by DDX11 helicase during DNA replication**

**Table of Contents:**

Appendix Fig. S1: CRISPR-Cas9 induced deletions in *TIMELESS* and *DDX11.*

Appendix Fig. S2: Backbone dynamics of Timeless 816-954.

Appendix Fig. S3: Expression of FLAG-Timeless and mutants.

Appendix Fig. S4: Association of DDX11 with chromatin bound PCNA before and after exposure to PDS.

Appendix Fig. S5: Expression of DDX11^KAK^ in *ddx11* cells.

Appendix Fig. S6: Immunofluorescence microscopy to quantitate γ-H2AX foci in wild type (WT), *ddx11* and *timeless* cells with and without exposure to PDS.

Appendix Table S1: Oligos used for molecular cloning, site-directed mutagenesis and CRISPR/Cas9 gene disruption.

Appendix Table S2: DNA sequences used in the DNA-binding experiments of Tim DBD and Timeless-Tipin.

Appendix Table S3: Data collection and refinement statistics for Tim DBD C-term (residues 883-947).

Appendix Table S4: Summary of the restraints used in the calculation of the Timeless 816-954 structure and characterisation of the ensemble of energy-minimised structures.

**Appendix Figures**


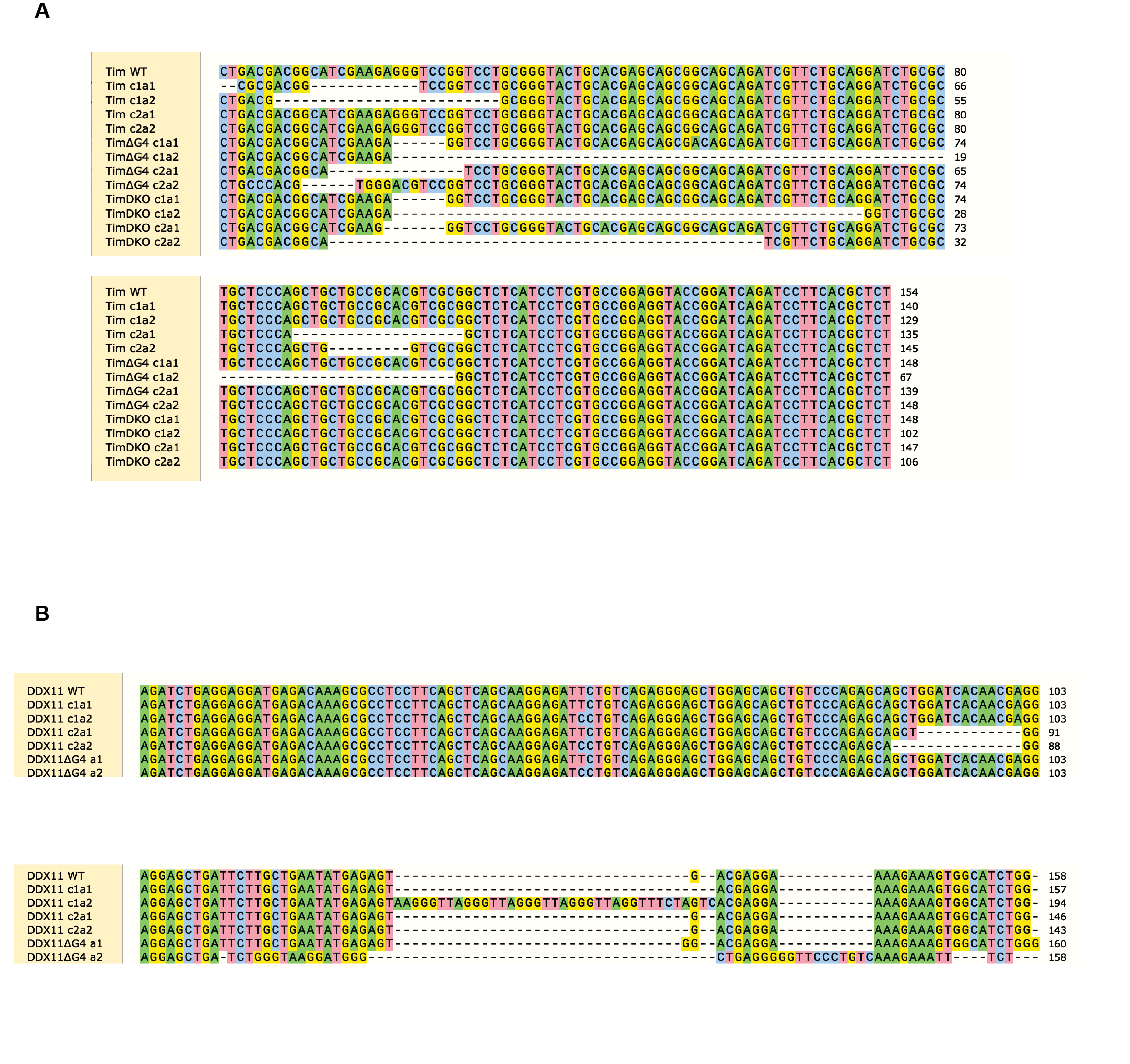


**Appendix Fig. S1. CRISPR-Cas9 induced deletions in *TIMELESS* and *DDX11.* A.** Aligned sequences of Exon 1 of the chicken *TIMELESS* locus showing the disruptions induced in the different clones used in this study. a1 and a2 refer to the two alleles. Note the telomeric repeat inserted in clone DDX11 c1a2. All disruptions lead to loss of frame. B. Aligned sequences of Exon 4 of the chicken *DDX11* locus showing the disruptions induced for the clones used in this study. All disruptions lead to loss of frame.


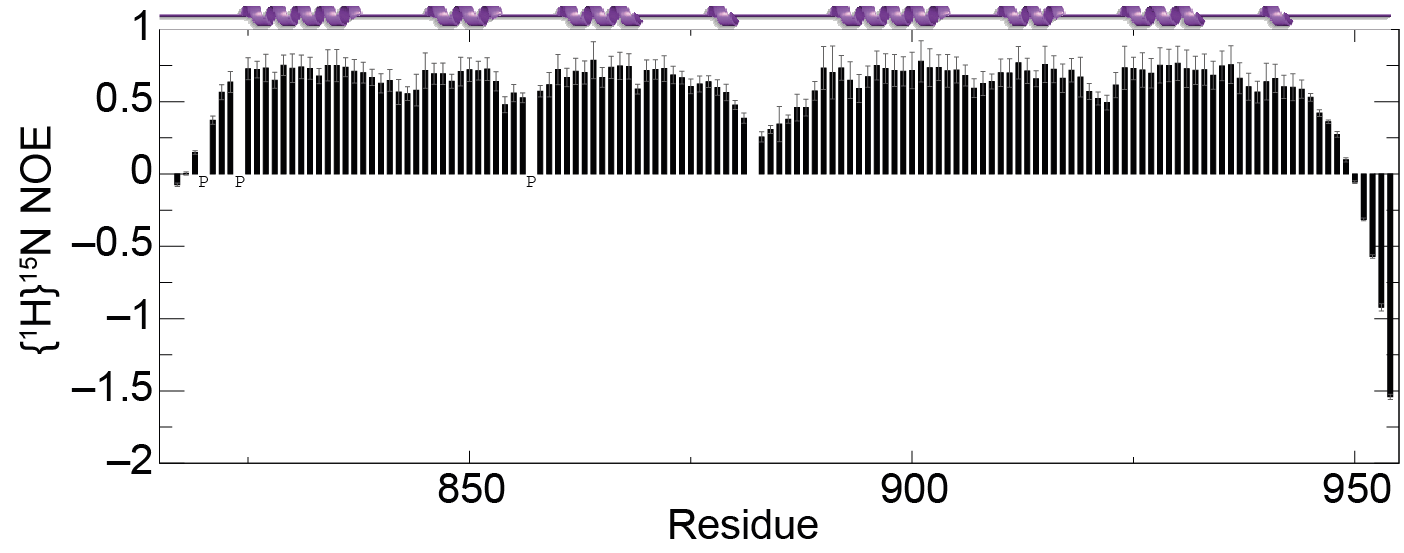


**Appendix Fig. S2. Backbone dynamics of Timeless 816-954.** (1H)^15^N heteronuclear nuclear Overhauser enhancement (NOE) values for the inter-domain linker region (residues 881-890) showed evidence of increased motions (NOE < 0.6) on a time scale faster than the overall tumbling rate. The linker is therefore flexible and confers independent mobility on the domains, which leads to a lack of global convergence of the NMR ensemble. Proline residues that lack ^1^H^N^ are marked ‘P’; the ^1^H^N^ of residue 882 was not detected due to conformational exchange broadening. Secondary structure from PDBsum.

**
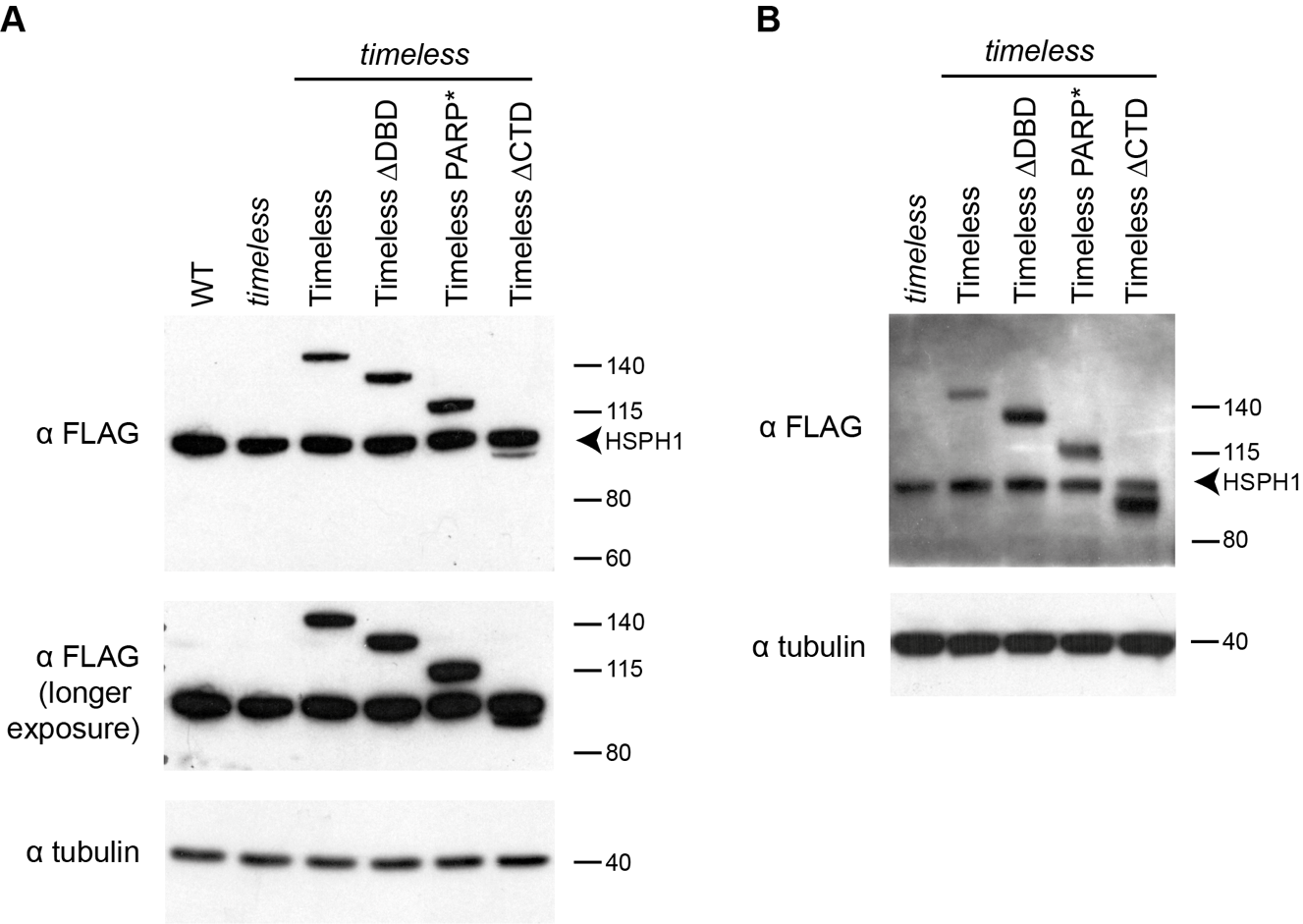
**

**Appendix Fig. S3. Expression of FLAG-Timeless and mutants.** FLAG-Timeless detected by Western blot in A. whole cell extracts and B. chromatin associated protein. Note chicken HSPH1, a heat-shock protein found in both cytoplasmic and chromatin compartments, contains a FLAG-like epitope in its C-terminus [820-DYKTEDMGEDDK] (Lara Phillips, PhD thesis, University of Cambridge, 2006).


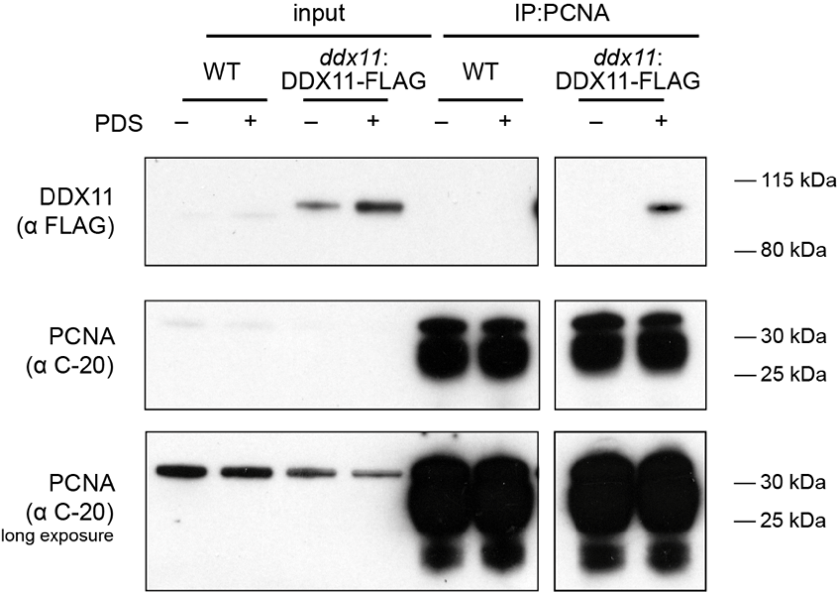


**Appendix Fig S4. Association of DDX11 with chromatin bound PCNA before and after exposure to PDS.** A biological repeat of the experiment shown in Figure 5A.

**
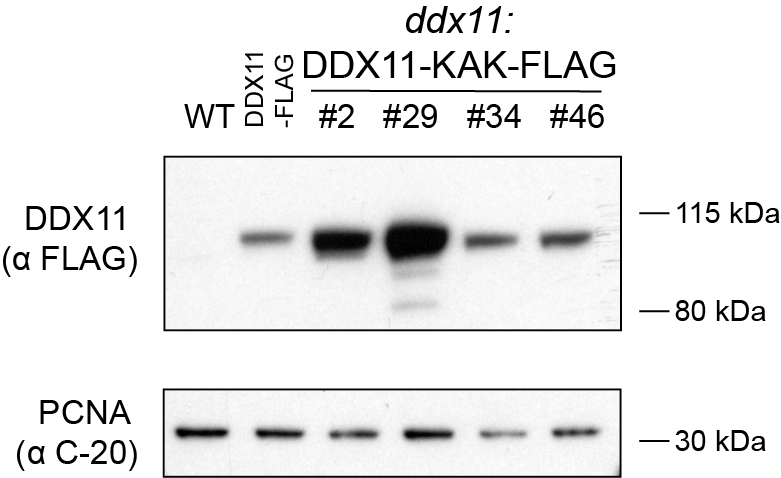
**

**Appendix Fig. S5. Expression of DDX11^KAK^ in *ddx11* cells.** Expression of DDX11^KAK^ (DDX11[E201K,Y202A,E203K]) (Cortone *et al.*, 2018) in four clones detected by western blotting of whole cell extracts for the FLAG epitope within the construct. Loading is controlled by blotting for PCNA. All four exhibited similar elevated levels of *BU-1* instability and the results were thus pooled in Figure 5C.


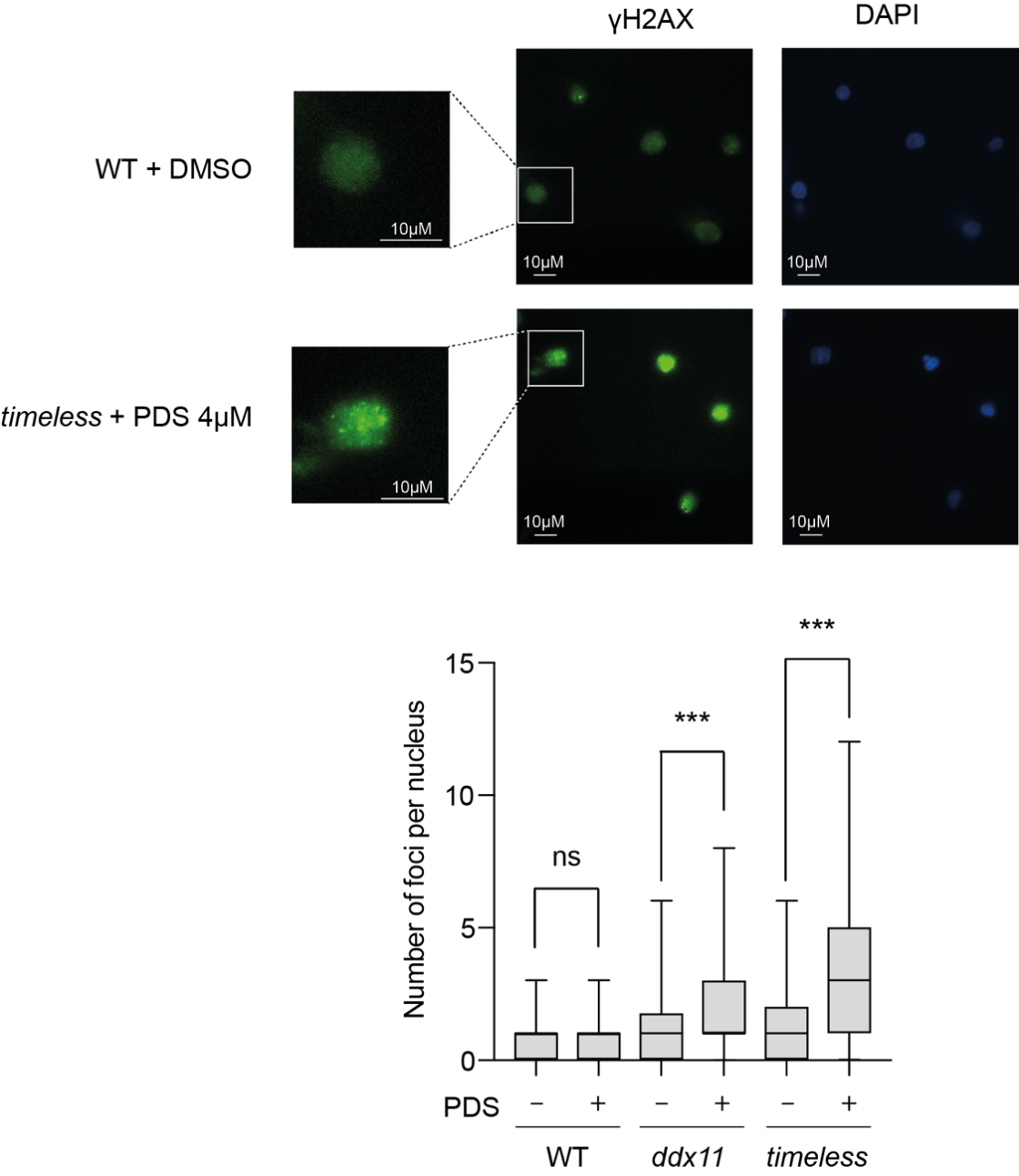


**Appendix Fig. S6. Immunofluorescence microscopy to quantitate γ-H2AX foci in wild type (WT), *ddx11* and *timeless* cells with and without exposure to PDS.** Cells were prepared exactly as for the flow cytometry experiments shown in Figure 6B. Foci were quantitated using FIJI (Schindelin *et al.*, 2012), as described in Methods. At high levels of γ-H2AX, the signal becomes pan-nuclear as illustrated in the lower right immunofluorescence panel. At least 50 cells were analysed for each condition. *** p < 0.001 Student’s t-test.

**Appdendix Tables**

**Appendix Table S1.** Oligos used for molecular cloning, site-directed mutagenesis and CRISPR/Cas9 gene disruption

| Identifier | 5′-3′ DNA Sequence |
| --- | --- |
| hTim∆DBD-D965-ClaI-GA-Fw | AGATGACTGAGGGCTATGGCTCCCTGGATGACAGGTCTTCCATCGATTTTTGCCAGGAAGATCTGGAAGAAGAGG |
| hTim∆DBD-S816-ClaI-GA-Rev | TTCCTCAGGCAGGTTTTCCTCTTCTTCCAGATCTTCCTGGCAAAAATCGATGGAAGACCTGTCATCCAGGGAGCC |
| hTimPBDtrunc-pcDNA3.1-bGH-TGA-NotI-GA-Fw | CAAGTCCAGGGTAGCTTAGTCTGAGCGGCCGCTCGAGTCTAGAGGGCCCTTCG |
| hTimPBDtrunc-NotI-GA Rev | CCCTCTAGACTCGAGCGGCCGCTCAGACTAAGCTACCCTGGACTTGTTCTGC |
| hTimC-tertrunc-pcDNA3.1-bGH-TGA-NotI-GA-Fw | GCTCCCTGGATGACAGGTCTTCCTGAGCGGCCGCTCGAGTCTAGAGGGCCCTTCG |
| hTimC-tertrunc-S816-NotI-GA-Rev | AAGGGCCCTCTAGACTCGAGCGGCCGCTCAGGAAGACCTGTTCATCCAGGGAGCC |
| hFANCJ K52R A155G Fw | AAGTAAGGCTAAGCTTCTTCCACTTCCTGTGGGAC |
| hDDX11-KAK-Fw | CTTTTTCTCCTCATCACTCTTGGCTTTGGCGAGGACCAGCTCCTCCT |
| hDDX11-KAK-Rev | AGGAGGAGCTGGTCCTCGCCAAAGCCAAGAGTGATGAGGAGAAAAAG |
| hFANCJ K52R A155G Rev | GTCCCACAGGAAGTGGAAGAAGCTTAGCCTTACTT |
| cDDX11 CRISPR gRNA top | CACCGTGCTGAATATGAGAGTGACG |
| cDDX11 CRISPR gRNA bottom | AAACCGTCACTCTCATATTCAGCAC |
| cTim CRISPR (KO) gRNA top | CACCGCGACGGCATCGAAGAGGGTC |
| cTim CRISPR (KO) gRNA bottom | AAACGACCCTCTTCGATGCCGTCGC |
| cTim CRISPR (C-ter truncation) gRNA top | CACCGACTTGTCGTCGTGTGCGGCC |
| cTim CRISPR (C-ter truncation) gRNA top | AAACGGCCGCACACGACGACAAGTC |

**Appendix Table S2.** DNA sequences used in the DNA-binding experiments of Tim DBD and Timeless-Tipin. The G4 DNA sequence used in Figure 2D is identical to the 1XAV sequence in Figure 3B, but with a Cy3 label at the 3’-end.

| **Identifier** | **5′-3′ DNA sequence (secondary-structure forming elements are highlighted in red)** |
| --- | --- |
|  | *DNA substrates for experiments in Figure 2D* |
| ssDNA | 6FAM-TGAGAGTGAGTAGAGTGAGTAA-Cy3 |
| dsDNA | ssDNA annealed ­with its complementary strand. |
| G4 DNA | TGAGGGTGGGTAGGGTGGGTAA-Cy3 |
|  | *DNA substrates for experiments in Figure 3A* |
| ss | 6FAM-ACGAGAGCTAGCACATTTTGAGTGTCAGTAGCGTCTGTAATTTTCACGTAGAACCTGT |
| ssHP | 6FAM-ACGAGAGCTAGCACATT**TTGAGGCTGCG**TTT**CGCAGCCTCAA**TTTCACGTAGAACCTGT |
| ssG4 | 6FAM-ACGAGAGCTAGCACATTTTGA**GGG**T**GGG**TA**GGG**T**GGG**TAATTTTCACGTAGAACCTGT |
|  | *DNA substrates for experiments in Figure 3B* |
| BU1 +3.5 (Schiavone *et al.*, 2014) | 6FAM-AGCTAGCACATTTTAA**GGG**CT**GGG**T**GGG**TGCTGTCAA**GGG**CT**GGG**TTTTCACGTAGAA |
| BU1 +3.5mut (Schiavone *et al.*, 2014) | 6FAM-AGCTAGCACATTTTAAGTTCTGTTTGTTTGCTGTCAAGTTCTGTTTTTTCACGTAGAA |
| 2JPZ (Dai *et al.*, 2007) | 6FAM-TTA**GGG**TTA**GGG**TTA**GGG**TTA**GGG**TT |
| 1XAV (Ambrus *et al.*, 2005) | 6FAM-TGA**GGG**T**GGG**TA**GGG**T**GGG**TAA |
| G4#2 (Schiavone *et al.*, 2014) | 6FAM-TAAT**GGG**TTT**GGG**TTT**GGG**TTT**GGG**T |
| G4#4 (Schiavone *et al.*, 2014) | 6FAM-TAATTTT**GGG**T**GGG**T**GGG**T**GGG**TTTT |
| 2O3M (Phan *et al.*, 2007) | 6FAM-TAA**GGG**A**GGG**CGCT**GGG**AGGA**GGG** |
| Bcl2Mid (Dai *et al.*, 2006) | 6FAM-ATA**GGG**CGC**GGG**AGGAAGG**GGG**C**GGG** |
| ρ-globin (Sarkies *et al.*, 2010) | 6FAM-TAA**GGGG**AGTAAAA**GGG**AGC**GGGG**TGCT**GGG** |

## Appendix Table S3. Data collection and refinement statistics for Tim DBD C-term (residues 883-947).

| *Data collection* |  |
| --- | --- |
| Wavelength | 0.9686 Å |
| Resolution range (Å) | 31.46 - 1.15 (1.191 - 1.15) |
| Space group | P6_5_ |
| Unit cell | 55.407 55.407 41.659 90 90 120 |
| Total reflections | 104424 (3064) |
| Unique reflections | 25072 (1920) |
| Multiplicity | 4.2 (1.6) |
| Completeness (%) | 96.33 (74.84) |
| Mean I/sigma(I) | 6.82 (1.36) |
| Wilson B-factor | 11.30 |
| R-merge | 0.1377 (0.4873) |
| R-meas | 0.1543 (0.6505) |
| R-pim | 0.06851 (0.4274) |
| CC1/2 | 0.975 (0.464) |
| CC* | 0.994 (0.796) |
| *Refinement* |  |
| Reflections used in refinement | 24985 (1922) |
| Reflections used for R-free | 1267 (101) |
| R-work | 0.1280 (0.2681) |
| R-free | 0.1548 (0.3632) |
| CC(work) | 0.973 (0.809) |
| CC(free) | 0.963 (0.769) |
| Number of non-hydrogen atoms | 659 |
| macromolecules | 527 |
| solvent | 132 |
| Protein residues | 62 |
| RMS(bonds) | 0.008 |
| RMS(angles) | 0.96 |
| Ramachandran favored (%) | 96.67 |
| Ramachandran allowed (%) | 3.33 |
| Ramachandran outliers (%) | 0.00 |
| Rotamer outliers (%) | 0.00 |
| Clashscore | 1.86 |
| Average B-factor | 17.72 |
| macromolecules | 14.14 |
| solvent | 32.01 |

Statistics for the highest-resolution shell are shown in parentheses.

**Appendix Table S4.** Summary of the restraints used in the calculation of the Timeless 816-954 structure and characterisation of the ensemble of energy-minimised structures.

| *NOE upper distance limits* |  | |
| --- | --- | --- |
| Total | 4730 | |
| Ambiguous | 1912 | |
| Unambiguous | 2818 | |
| *Dihedral angle constraints* |  | |
| Total | 278 | |
| *Residual NOE violation*s (Å) |  | |
| Number ≥ 0.5 | 0 | |
| Number ≥ 0.1 | 117 | |
| *Residual angle violations* (deg.) |  | |
| Number ≥ 2.0 | 0 | |
| *Energies* (kcal mol^–1^) |  | |
| Total | –4691 ± 40 | |
| van der Waals | –1348 ± 40 | |
| Electrostatic | –5207 ± 53 | |
| *Ramachandran statistics (% residues)* |  | |
| Core regions | 83.7 | |
| Allowed regions | 16.3 | |
| Generously allowed regions | 0 | |
| Disallowed regions | 0 | |
| *r.m.s.d. from ideal geometry* |  | |
| Bond lengths (Å) | 0.0052 ± 0.0002 | |
| Bond angles (deg.) | 0.660 ± 0.008 | |
| *r.m.s.d. to mean coordinates* (Å) | *Backbone* | *Heavy atoms* |
| Protein (824-880) | 0.494 | 0.580 |
| Protein (891-944) | 0.487 | 0.527 |
| For the analysis, 20 water-refined, energy-minimised structural conformations were used. Ramachandran statistics were calculated using PDBsum. | | |

**Supplementary References**

Ambrus A, Chen D, Dai J, Jones RA, Yang D (2005) Solution structure of the biologically relevant G-quadruplex element in the human c-MYC promoter. Implications for G-quadruplex stabilization. *Biochemistry*, **44:** 2048–2058

Cortone G, Zheng G, Pensieri P, Chiappetta V, Tatè R, Malacaria E, Pichierri P, Yu H, Pisani FM (2018) Interaction of the Warsaw breakage syndrome DNA helicase DDX11 with the replication fork-protection factor Timeless promotes sister chromatid cohesion. *PLoS Genet*, **14:** e1007622

Dai J, Carver M, Punchihewa C, Jones RA, Yang D (2007) Structure of the Hybrid-2 type intramolecular human telomeric G-quadruplex in K+ solution: insights into structure polymorphism of the human telomeric sequence. *Nucleic Acids Res*, **35:** 4927–4940

Dai J, Chen D, Jones RA, Hurley LH, Yang D (2006) NMR solution structure of the major G-quadruplex structure formed in the human BCL2 promoter region. *Nucleic Acids Res*, **34:** 5133–5144

Phan AT, Kuryavyi V, Burge S, Neidle S, Patel DJ (2007) Structure of an unprecedented G-quadruplex scaffold in the human c-kit promoter. *J Am Chem Soc*, **129:** 4386–4392

Sarkies P, Reams C, Simpson LJ, Sale JE (2010) Epigenetic instability due to defective replication of structured DNA. *Mol Cell*, **40:** 703–713

Schiavone D, Guilbaud G, Murat P, Papadopoulou C, Sarkies P, Prioleau M-N, Balasubramanian S, Sale JE (2014) Determinants of G quadruplex-induced epigenetic instability in REV1-deficient cells. *The EMBO Journal*, **33:** 2507–2520

Schindelin J, Arganda-Carreras I, Frise E, Kaynig V, Longair M, Pietzsch T, Preibisch S, Rueden C, Saalfeld S, Schmid B, Tinevez J-Y, White DJ, Hartenstein V, Eliceiri K, Tomancak P, Cardona A (2012) Fiji: an open-source platform for biological-image analysis. *Nature Methods*, **9:** 676–682
