# Supplementary material for: Timeless couples G‐quadruplex detection with processing by DDX11 helicase during DNA replication
Source: EMBO J. 2020 Jul 23;39(18):e104185. doi: 10.15252/embj.2019104185 (PMC7506991; doi:10.15252/embj.2019104185)
Supplement: Supplementary file 2 — Expanded View Figures PDF [file EMBJ-39-e104185-s002.pdf]

Expanded View Figures

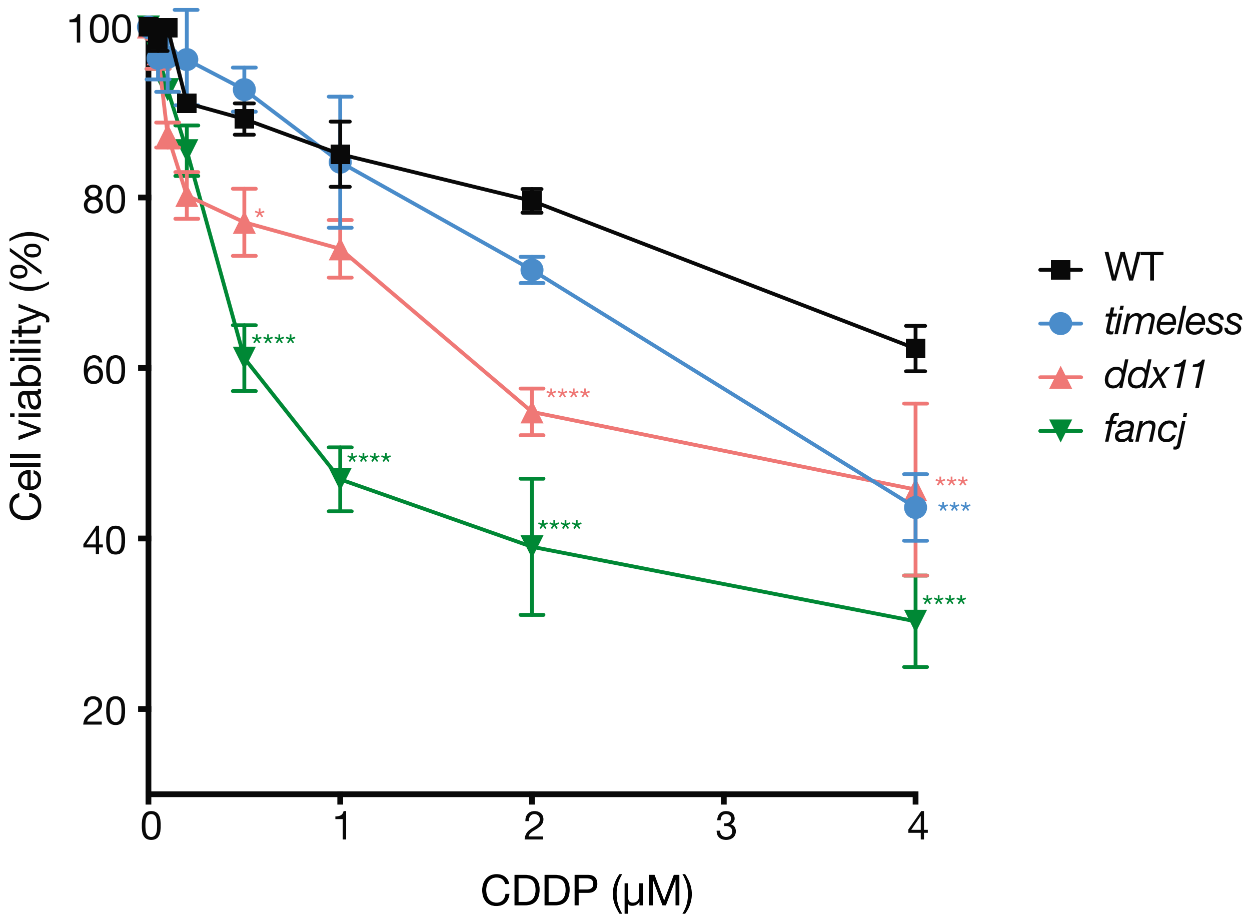

Figure EV1. Sensitivity of wild type (WT), *timeless*, *ddx11* and *fancj* DT40 mutants to cisplatin (CDDP).

Cell viability, assessed by MTS assay, of DT40 wild type, *ddx11*, *timeless* and *fancj*, after 72 h in presence of cisplatin at the indicated doses. The values represent the means (error bars indicate SD) of two independent experiments performed in triplicate. *$P < 0.05$, ***$P < 0.001$ and ****$P < 0.0001$; one-way ANOVA compared to the wild type.

© 2020 MRC Laboratory of Molecular Biology

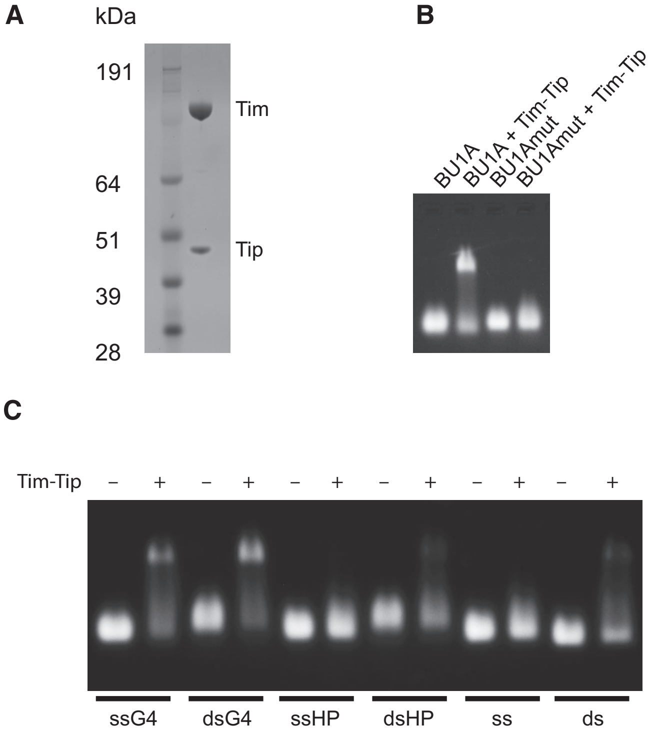

Figure EV2. The Timeless–Tipin complex shows a preference for binding G-quadruplex DNA structures.

A Coomassie-stained SDS–PAGE gel of purified Timeless–Tipin complex.

B Electrophoretic mobility shift assay (EMSA) showing the binding of Timeless–Tipin to G-quadruplex sequence BU1A + 3.5. Mutation of the G-quadruplex sequence (BU1A + 3.5 mut) disrupts Timeless–Tipin binding (see Appendix Table S2 for sequence details). Timeless–Tipin and DNA are both present at a final concentration of 5 μM.

C EMSA showing the binding of Timeless–Tipin to G-quadruplex sequences (ssG4, dsG4) but not single-stranded DNA (ss), double-stranded DNA (ds) or hairpin-containing sequences (ssHP, dsHP). Timeless–Tipin and DNA are both present at a final concentration of 5 μM.

© 2020 MRC Laboratory of Molecular Biology

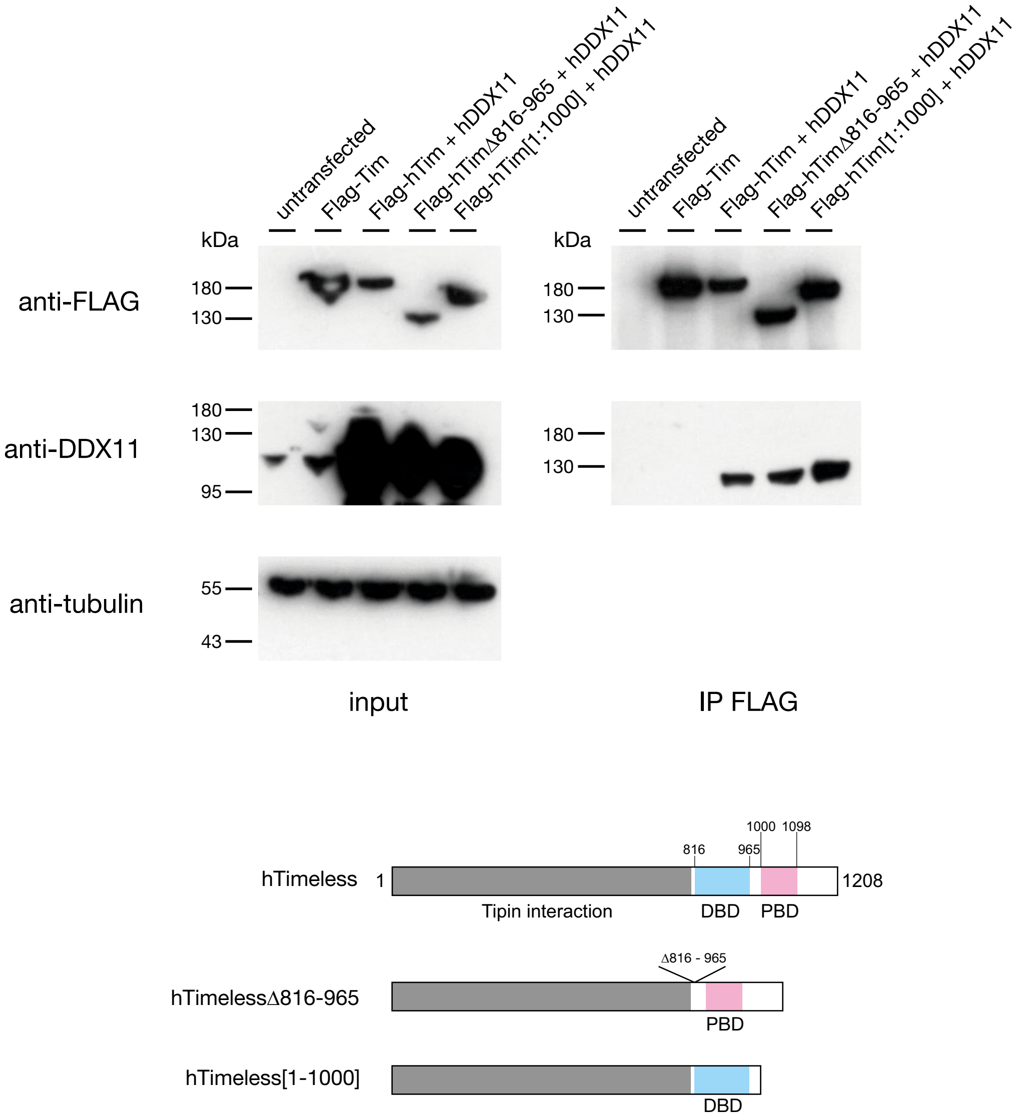

Figure EV3. The C-terminus of Timeless is not required for its interaction with DDX11.

HEK293T cells were transiently transfected with a plasmid encoding Flag-hTimeless, or co-transfected with plasmids encoding hDDX11 and Flag-Timeless or with Timeless mutated to delete the DNA-binding domain (ΔDBD: deletion of region S816–S965) or PARP-binding domain (PARP*: truncation at V1000). Twenty-four h after transfection, whole-cell extracts were subjected to immunoprecipitation with anti-Flag magnetic beads. Western Blot analyses were performed to detect overexpressed DDX11 protein in the pulled down samples using a specific antibody. Upper panel: Input and pulled down samples transfected with different Timeless constructs detected with an anti-Flag antibody. Bottom panel: Input and pulled down samples transfected with different Timeless constructs detected with an anti-DDX11 antibody. Tubulin was used as a loading control for the input samples.

© 2020 MRC Laboratory of Molecular Biology

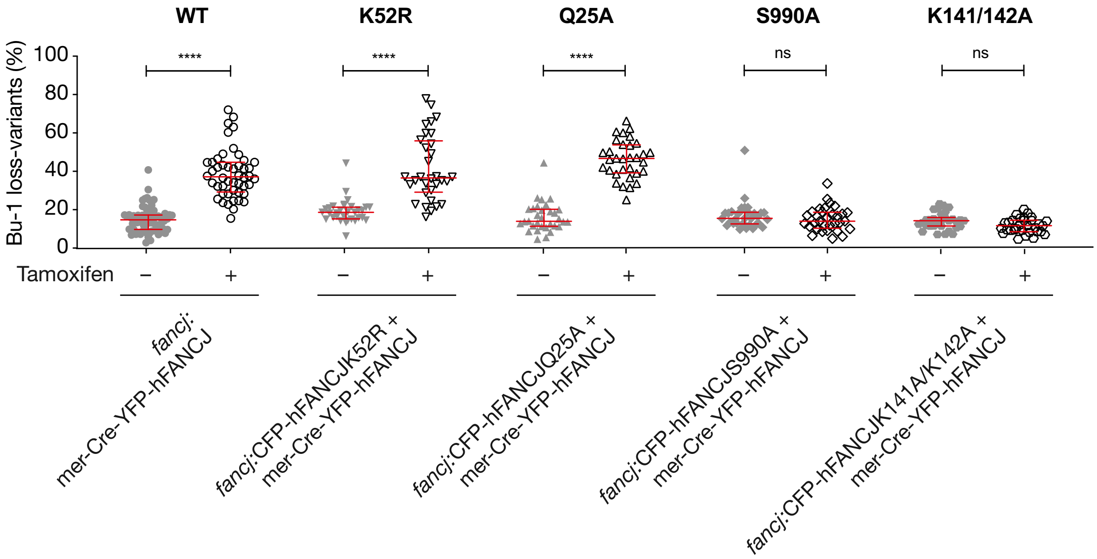

Figure EV4. The catalytic activity of FANCJ is required for its role in suppressing G4-induced instability of *BU-1* expression.

Fluctuation analysis for the generation of Bu-1 loss variants in an inducible system to study FANCJ function (see Materials and Methods for full details). Briefly, FANCJ-deficient DT40 cells are rescued with two transgenes, one encoding the wild-type protein and the other the mutant in question. The wild-type transgene is flanked by loxP sites and can be deleted by expression of Cre recombinase, induced by treatment of cells with tamoxifen. The K52R and Q25A mutants of FANCJ both disrupt the helicase activity of the enzyme (Cantor *et al*, 2001; Wu *et al*, 2012b). S990A disrupts the interaction of FANCJ with BRCA1, which is important for the role of FANCJ in homologous recombination (Xie *et al*, 2010). K141/142A disrupts the interaction of FANCJ with MutLα, which is needed for efficient interstrand crosslink repair (Peng *et al*, 2007). The data for each cell line represent the pooled results of at least two independent fluctuation analyses with a minimum of 32 data points per condition. Bars and whiskers represent median and interquartile range, respectively. ****$P < 0.0001$; one-way ANOVA for comparison between uninduced and tamoxifen-induced lines.

 © 2020 MRC Laboratory of Molecular Biology

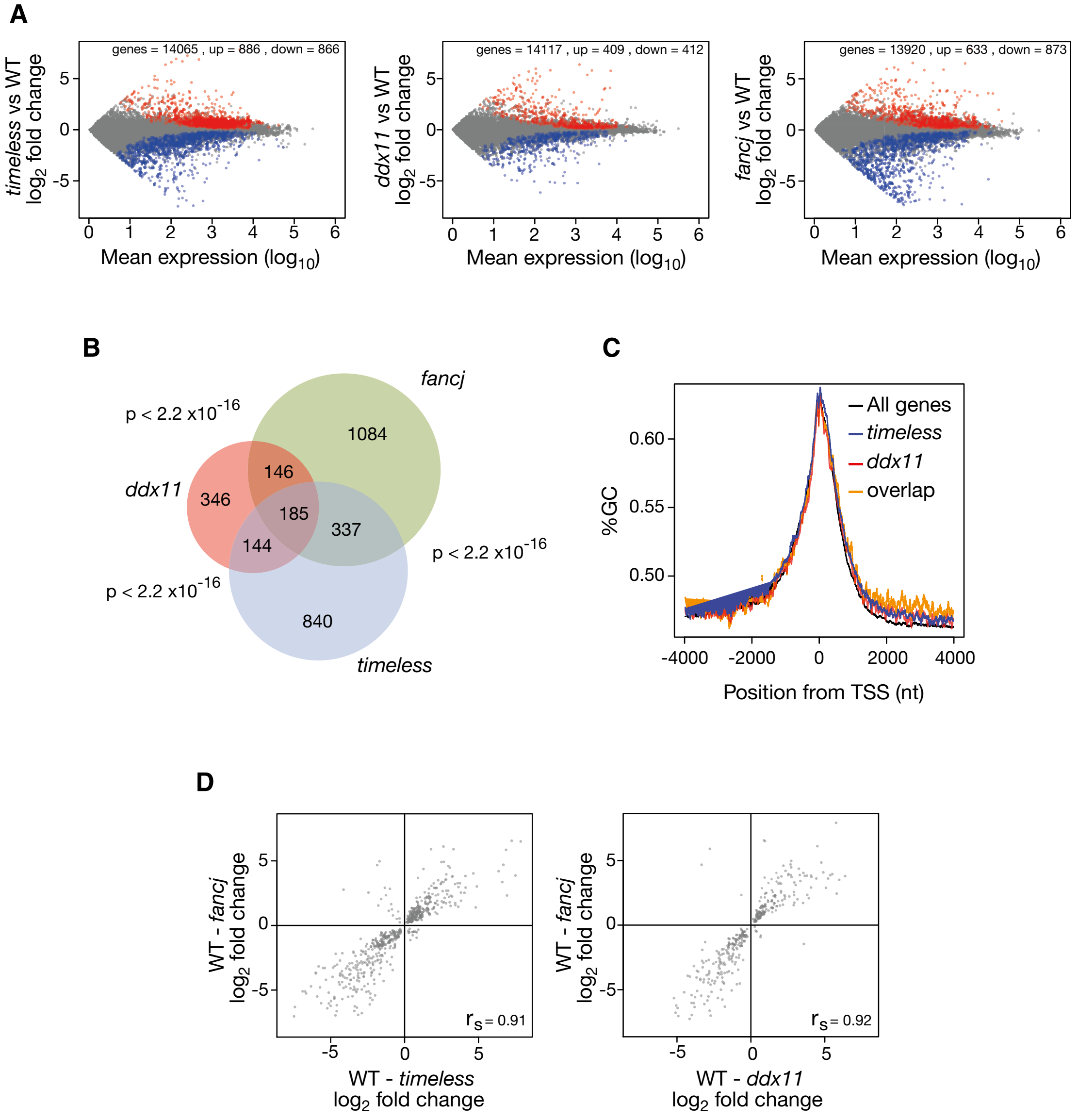

Figure EV5. Gene expression dysregulation in *timeless* and *ddx11* DT40 cells.

A Dysregulated genes in *timeless* (left panel), *ddx11* (centre panel) and *fancj* (right panel) mutants relative to wild type. All genes with > 1 transcript per million in both conditions are plotted. Significantly ($P \geq 0.95$) upregulated genes shown in red; downregulated in blue.

B Venn diagram showing the overlap in genes deregulated in *timeless*, *ddx11* and *fancj* relative to wild type. $P < 2.2 \times 10^{-16}$ for each pairwise comparison (Fisher hypergeometric distribution).

C GC content around the TSS in genes dysregulated in *timeless* (blue), *ddx11* (red) and in both mutants ("overlap", orange) compared with all genes (black).

D Correlation of magnitude and direction of change of genes dysregulated (relative to wild type) in *fancj us. timeless* (left panel) and *ddx*11 (right panel) DT40 cells. r_s (Spearman rho) is shown for each correlation.

© 2020 MRC Laboratory of Molecular Biology
